# Reshaping the Ventricle From Within: MIRTH (Myocardial Intramural Remodeling by Transvenous Tether) Ventriculoplasty in Swine

**DOI:** 10.1016/j.jacbts.2022.07.002

**Published:** 2022-11-09

**Authors:** Christopher G. Bruce, Jaffar M. Khan, Toby Rogers, D. Korel Yildirim, Andrea E. Jaimes, Felicia Seemann, Marcus Y. Chen, Kendall O’Brien, Daniel A. Herzka, William H. Schenke, Michael A. Eckhaus, Amanda G. Potersnak, Adrienne Campbell-Washburn, Vasilis C. Babaliaros, Adam B. Greenbaum, Robert J. Lederman

**Affiliations:** a Cardiovascular Branch, Division of Intramural Research, National Heart Lung and Blood Institute, National Institutes of Health, Bethesda, Maryland, USA; b MedStar Washington Hospital Center, Washington, DC, USA; c Division of Veterinary Resources, National Institutes of Health, Bethesda, Maryland, USA; d Structural Heart and Valve Center, Emory University Hospital, Atlanta, Georgia, USA

**Keywords:** cardiac repair, cardiomyopathy, heart failure/etiology/mortality/surgery, surgical ventricular restoration, ventricular remodeling, ventriculoplasty, CMR, cardiac magnetic resonance, CTO, chronic total occlusion, EDEN, electrocardiographic radial depth navigation, EDV, end-diastolic volume, Ees, end-systolic elastance, ESPVR, end-systolic pressure-volume relationship, ESV, end-systolic volume, LVEDP, left ventricular end-diastolic pressure, LVESP, left ventricular end systolic pressure, MIRTH, myocardial intramural remodeling by transvenous tether, PRSW, preload recruitable stroke work, PVA, pressure-volume area

## Abstract

MIRTH (Myocardial Intramural Remodeling by Transvenous Tether) is a transcatheter ventricular remodeling procedure. A transvenous tension element is placed within the walls of the beating left ventricle and shortened to narrow chamber dimensions. MIRTH uses 2 new techniques: controlled intramyocardial guidewire navigation and EDEN (Electrocardiographic Radial Depth Navigation). MIRTH caused a sustained reduction in chamber dimensions in healthy swine. Midventricular implants approximated papillary muscles. MIRTH shortening improved myocardial contractility in cardiomyopathy in a dose-dependent manner up to a threshold beyond which additional shortening reduced performance. MIRTH may help treat dilated cardiomyopathy. Clinical investigation is warranted.

## Highlights

• MIRTH is a novel catheter-based intramyocardial ventricular remodeling procedure.
• Intramyocardial guidewire navigation is a new technique that enables MIRTH, allowing the operator to easily steer an angioplasty guidewire to any location within the ventricular walls.
• Intramyocardial guidewire navigation is assisted by EDEN, a novel tool that increases procedural accuracy and speed by ensuring the operator of a midmyocardial guidewire position when performing MIRTH.
• MIRTH reduces left ventricular chamber dimensions and, by the principles of the law of Laplace, wall stress.
• MIRTH causes a dose-dependent improvement in measures of left ventricular performance (increased contractility, increased ventricular mechanical efficiency, and reduced myocardial oxygen demand) in cardiomyopathic animals.

## Visual Abstract

**Figure:**
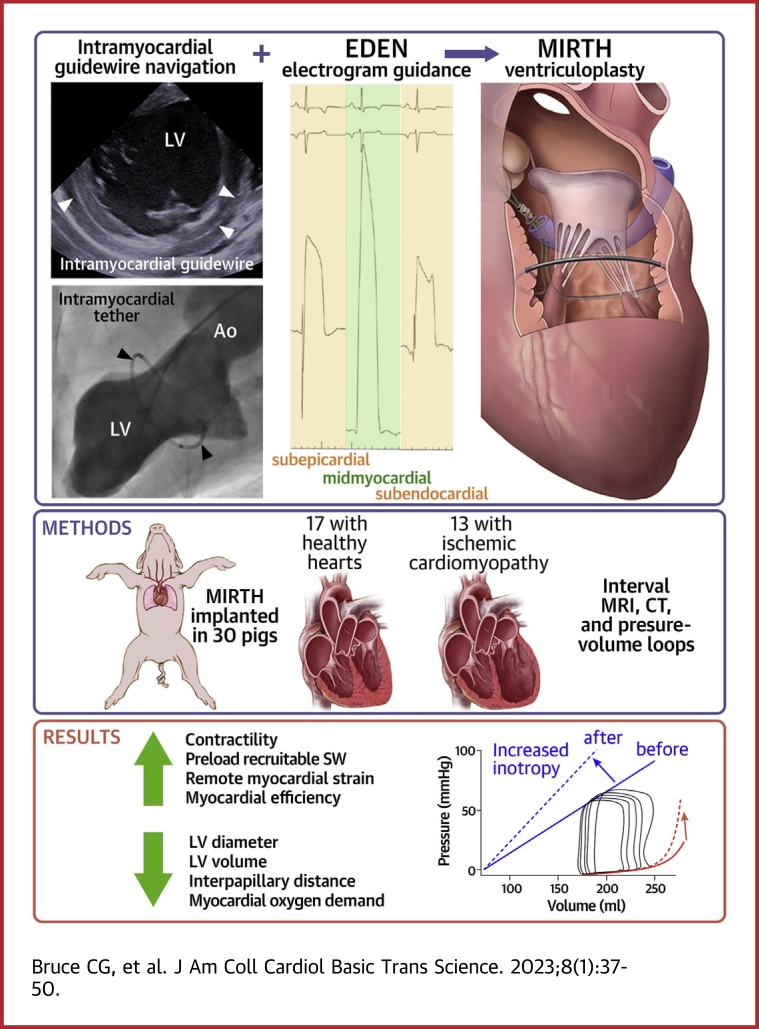

Myocardial remodeling devices aim to treat heart failure and enhance ventricular mechanical efficiency by reducing chamber size and wall stress according to Laplace principles. Numerous surgical and transcatheter global and segmental remodeling strategies^1^, ^2^, ^3^, ^4^, ^5^, ^6^, ^7^, ^8^, ^9^, ^10^ suffer limitations such as surgical morbidity, right ventricular restriction, or endocardial anchor failure. We developed a transcatheter procedure to overcome these limitations.

We engineered an approach to narrow the left ventricle circumferentially by implanting an intramyocardial tension element percutaneously within the ventricular walls using commercial off-the-shelf devices. This required new catheter techniques to navigate an extravascular guidewire within the heart muscle while remaining within the endocardial and epicardial boundaries in a procedure called MIRTH (Myocardial Intramural Remodeling by Transvenous Tether).

We hypothesized that in a large-mammal model: 1) transvenous catheter tools can be directed purposefully within beating ventricular myocardium; 2) local electrograms indicating midmyocardial position can ensure the navigating guidewire remains between endocardial and epicardial boundaries; 3) basal- or midmyocardial-level MIRTH loops can narrow corresponding left ventricular regions in naive swine; 4) MIRTH navigation can be accomplished in naive and fibrotic myocardium; and 5) MIRTH traction exhibits a dose-response relationship on myocardial geometry and function.

## Methods

### Animals

Animal experiments were approved by the institutional animal use and care committee and followed contemporary National Institutes of Health guidelines. Juvenile Yorkshire swine (40-53 kg) were pretreated with amiodarone and underwent general anesthesia, mechanical ventilation, percutaneous jugular venous and femoral arterial access, heparin anticoagulation, continuous invasive hemodynamic monitoring, and cefazolin prophylaxis.

After technical development (Supplemental Methods), MIRTH implants were performed on 33 animals, 17 with healthy myocardium and 16 with ischemic cardiomyopathy. We tested feasibility and safety in healthy animals, with intentional overshortening to exaggerate erosive stress in survival experiments. Because it is difficult to improve cardiac performance in healthy animals, we created an infarct model of cardiomyopathy. Myocardial infarction was produced via multivessel (typically mid–left anterior descending and obtuse marginal branches) transcoronary ethanol chemoablation followed by survival for 4 to 12 weeks. This model, which has approximately 70% spontaneous mortality by 90 days in our laboratory,^11^ also allowed us to test MIRTH through fibrotic myocardium.

### MIRTH concept

A guidewire penetrates the myocardium from the proximal coronary sinus into the heart base, is directed to encircle the left ventricle along a “short-axis” trajectory between endocardial and epicardial walls, is retrieved and externalized, and then is exchanged for a tensioning element that is shortened to achieve the desired geometry and then locked in place.

### Detailed MIRTH technique

**Figure 1:**
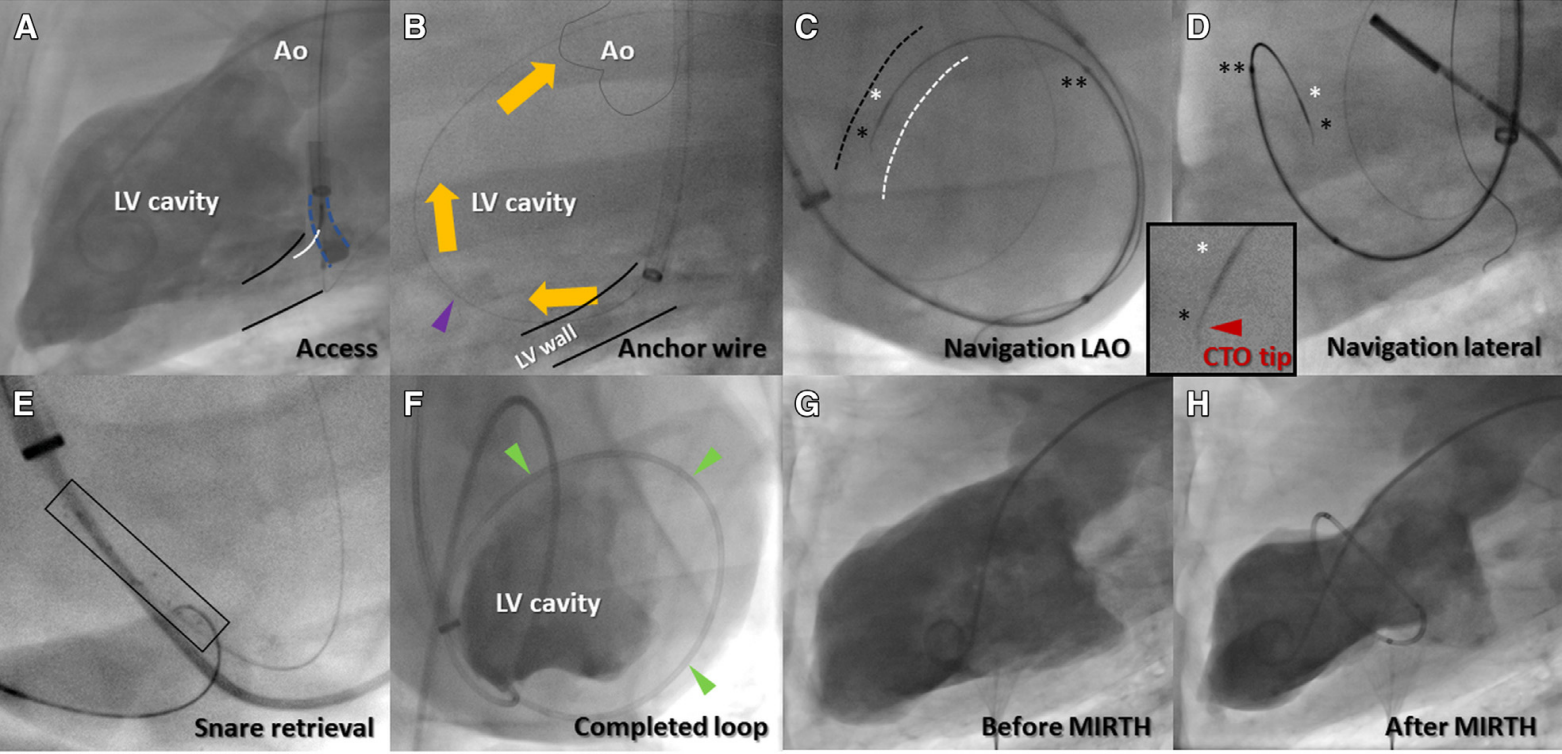
Representative MIRTH Procedure **(A)** The posterobasal left ventricular myocardium **(black outline)** is accessed directly from the coronary sinus **(interrupted blue outline)** using a needle catheter **(white overlay)**. **(B)** An “anchor” guidewire **(purple arrow)** is advanced through the myocardium into the left ventricular cavity where it is ensnared and externalized **(orange arrows)**. This stabilizes subsequent catheters. **(C and D)** The MIRTH (Myocardial Intramural Remodeling by Transvenous Tether) “navigation” guidewire with chronic total occlusion (CTO) tip **(single black asterisk and inset)** is steered around the ventricle, ensheathed by coaxial 0.014-inch **(white asterisk)** and 0.035-inch **(double asterisk)** microcatheters and guided by fluoroscopy and electrocardiographic radial depth navigation electrograms. Approximate locations of left ventricular (LV) **(white dash)** and right ventricular **(black dash)** septal endocardial borders added for orientation. **(E)** A self-expanding stent retriever **(black rectangle)** is advanced over the anchor guidewire within the posterior basal myocardium to act as a target and snare for the returning MIRTH navigation guidewire. **(F)** A completed MIRTH loop within the walls of the left ventricle **(green arrows)**, viewed from apex of the heart, equivalent to a human LAO caudal projection. **(G and H)** Baseline- and postprocedure LV angiograms of a midventricular-level MIRTH. The traversing guidewire was exchanged for suture and implant and secured and cut with a surgical crimp fastener. Ao = aorta; LAO = left anterior oblique.

**Figure 2:**
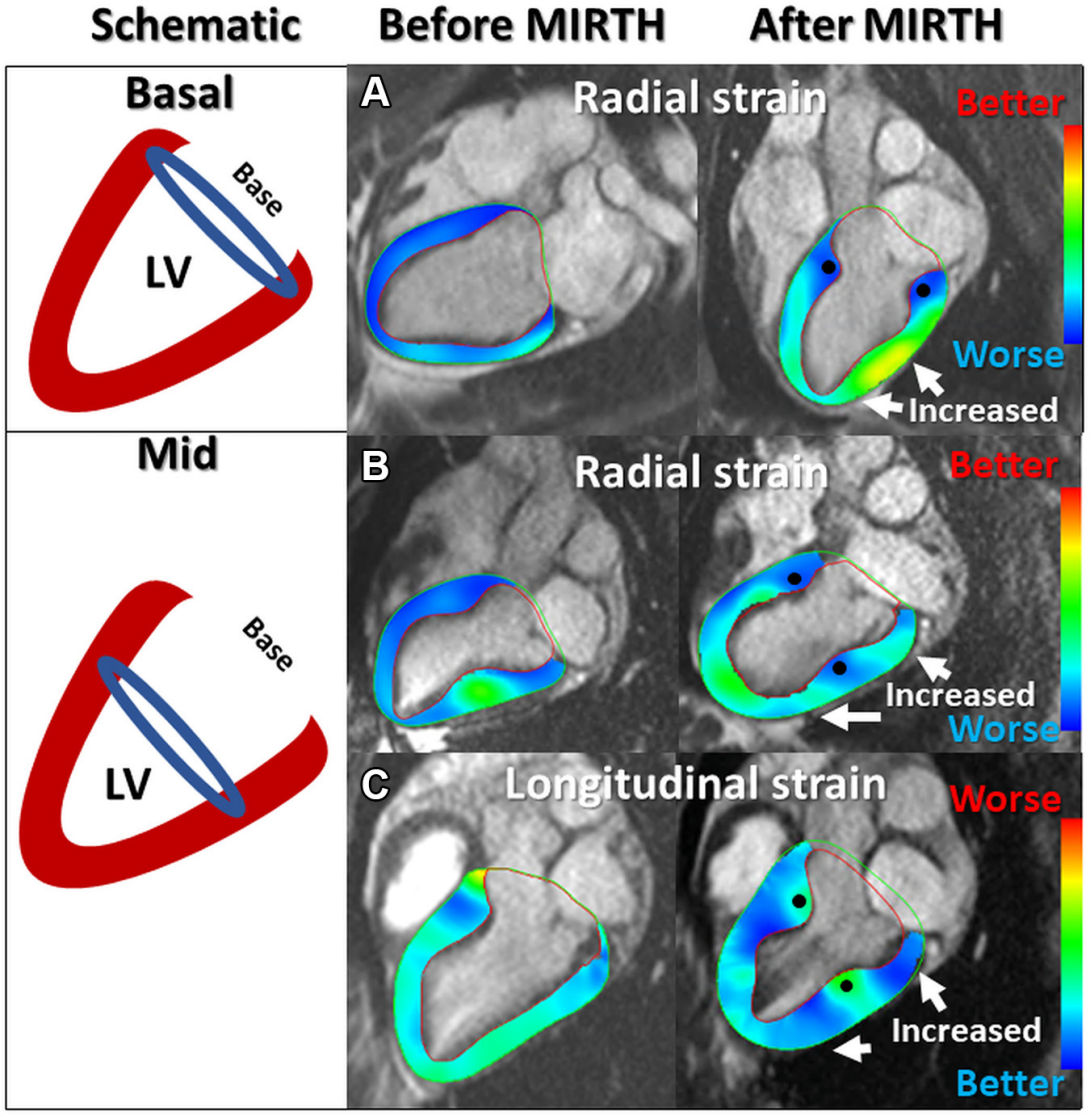
Basal and Midventricular MIRTH in Healthy Animals **(Left)** A schematic showing implant location for **(upper)** basal and **(lower)** midventricular MIRTH. **(Right) (A)** Radial strain before and after a basal MIRTH implant. Strain is decreased at the implant **(blue)** and increased **(green-yellow)** in the midventricle and apex **(arrows)**. **(B)** Radial strain before and after midventricular MIRTH. The implant decreased local strain **(blue)** and caused increased strain **(green)** in remote basal and apical myocardium **(arrows)**. **(C)** Longitudinal strain after midventricular MIRTH. The implant decreased local strain **(green)** and increased **(blue)** remote strain at the base and apex **(arrows)**. **Solid circle** identifies the intramyocardial implant location. Abbreviations as in Figure 1.

**Figure 3:**
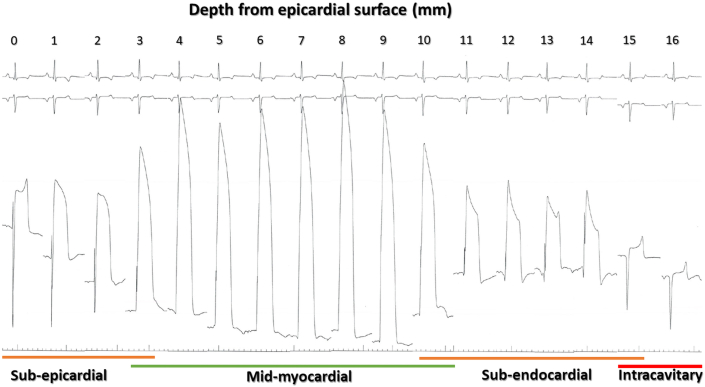
EDEN Electrogram Morphology to Guide Intramyocardial Guidewire Navigation Depth is shown in millimeters from the epicardial surface. The injury current (ST-segment elevation subsuming the QRS complex) exhibits a biphasic pattern during wire advancement from the epicardial toward the endocardial surface, gradually increasing to a maximum in the midmyocardium. The ST-segment elevation then declines with additional advancement toward the endocardium. The injury current disappears when the guidewire tip enters the left ventricular chamber at 16-mm. Color-coded regions indicate how operators interpret electrocardiographic radial depth navigation (EDEN) electrograms during navigation: deep myocardial **(green)**, subendocardial and subepicardial **(orange)**, and intracavity **(red)**.

**Figure 4:**
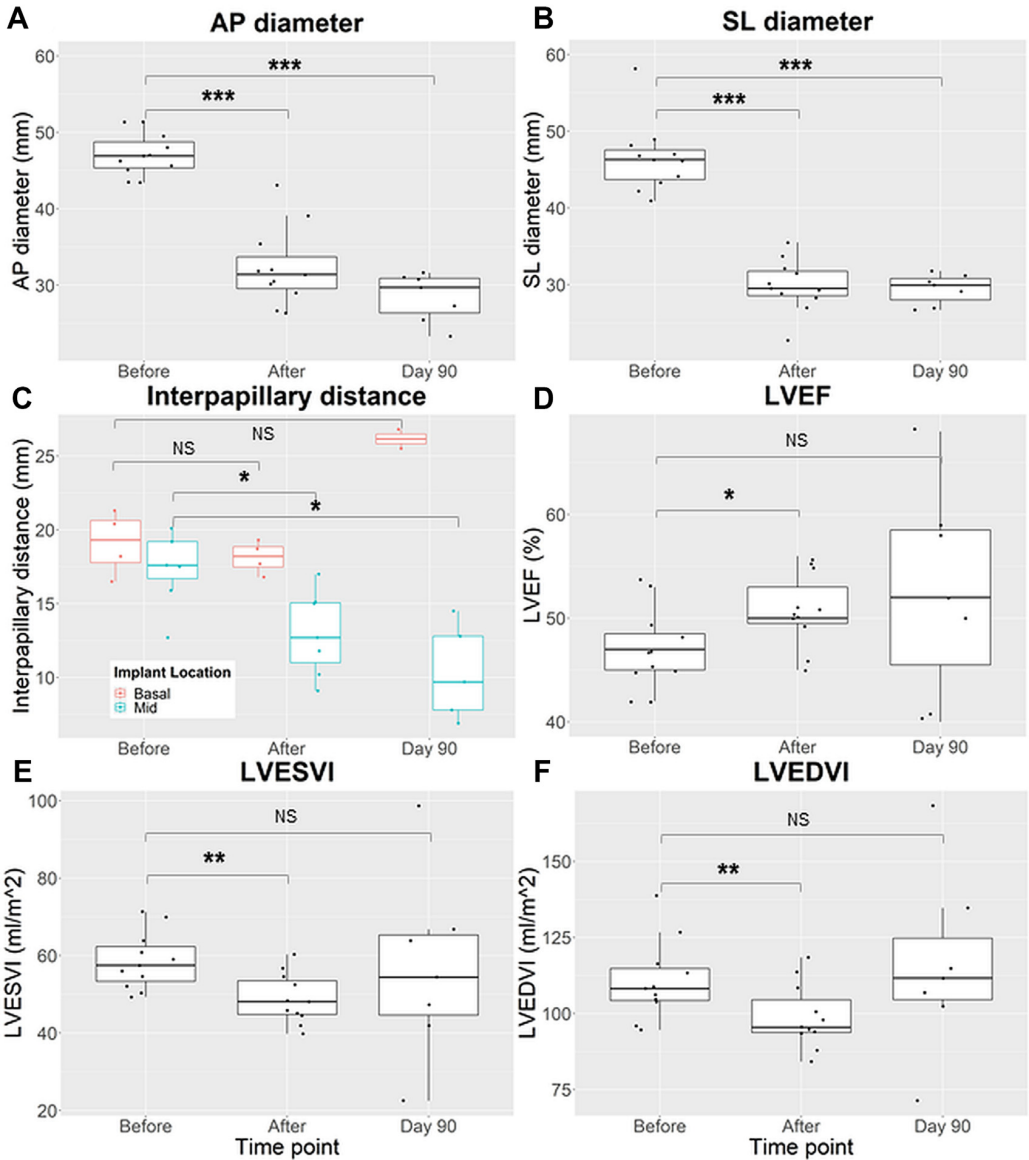
MIRTH-Induced Changes in Left Ventricular Geometry, Volume, and Ejection Fraction Over 90 Days in Healthy Animals **(A and B)** Left ventricular anteroposterior (AP) and septolateral (SL) diameters at the level of MIRTH implant. **(C)** The impact of basal and midventricular MIRTH (Myocardial Intramural Remodeling by Transvenous Tether) on the interpapillary distance. **(D to F)** Left ventricular ejection fraction (LVEF) and indexed left ventricular volumes. Data were compared using Wilcoxon signed rank tests. ∗*P <* 0.05, ∗∗*P <* 0.01, ∗∗∗*P <* 0.001. LVEDVI = indexed left ventricular end-diastolic volume; LVESVI = indexed left ventricular end systolic volume.

To conduct MIRTH, a transvenous catheter is first positioned in the coronary sinus to deliver a needle catheter (Pioneer Plus, Philips) into the posterobasal left ventricular myocardium (Figure 1A, Video 1) and deliver a stiff 0.014-inch guidewire (AstatoXS 20, Asahi-Intecc) through the myocardium and into the left ventricular chamber where it is ensnared (EnSnare 18/30, Merit Medical) via a retrograde transaortic catheter to serve as a position anchor (Figure 1B, Video 1). The floor of the coronary sinus (posterobasal left ventricular wall) is balloon dilatated to create space for later ensnarement within the myocardial wall. The MIRTH trajectory is navigated using a 0.014-inch guidewire indicated for chronic total occlusion (CTO) traversal (AstatoXS 20) with a 1-mm 30° to 40° “CTO tip”^12^ and a coaxial microcatheter (Figures 1C and 1D, Videos 2 and 3). The MIRTH guidewire is delivered to the myocardium using the parallel wiring technique and steered around the ventricle at a preselected basal or midventricular base-to-apex level (Figure 2). The MIRTH guidewire is navigated using biplane fluoroscopy and continuous electrocardiographic radial depth navigation (EDEN) electrograms (described later). EDEN electrograms indicate when the MIRTH guidewire needs redirection away from endocardial or epicardial borders. After encircling the ventricle, the MIRTH guidewire is steered into, ensnared, and externalized by a self-expanding stent retriever (Solitaire Platinum, EV3) snare prepositioned within the predilatated posterobasal ventricular wall (Figure 1E, Video 1), and the anchor guidewire is withdrawn. The resulting venovenous guidewire rail (Figure 1F) is exchanged for ultra-high molecular weight polyethylene braided suture (HS Fiber, Riverpoint Medical) protected by a segment of cardiac magnetic resonance (CMR)-compatible 316L braided microcatheter (NaviCross, Terumo) cut to the intended circumference (Video 2). Applied tension was locked with a surgical crimp fastener (Cor-Knot, LSI Solutions), and all catheters were then removed (Figures 1H, 3, and 4, and Videos 2 and 3).

### EDEN

We characterized unipolar intramyocardial electrogram patterns that reflected relative intramyocardial depth (Supplemental Methods). Electrograms were recorded continuously during MIRTH guidewire navigation by exposing only the guidewire tip using the 0.014-inch microcatheter as insulation^13^ and connecting the proximal guidewire to a precordial electrode on the hemodynamic recording system (Sensis, Siemens).

### Imaging and energetics (pressure-volume loops)

Procedures were guided by biplane fluoroscopy (Artis Zee, Siemens) with projection angles selected by cone-beam computed tomography (DynaCT, Siemens), EDEN electrograms (described later), and right-sided intracardiac echocardiography (investigational 2-dimensional intracardiac echocardiography, nonhuman and noncommercial, Siemens).

Cardiac function was assessed using CMR at 0.55-T (Prototype MAGNETOM Aera, Siemens).^14^ The interpapillary distance was the center of mass between the papillary endocardial surfaces at end-systole on short-axis CMR.

Dynamic pressure-volume loops were calibrated using CMR end-systolic and end-diastolic volumes and measured using conductance catheters (Sigma-M, CD Leycom) during balloon caval occlusion.^15^^,^^16^ The end-systolic pressure-volume relationship (ESPVR), V_0_ (x-intercept of linear ESPVR regression), end-diastolic pressure-volume relationship slope (of the linear end-diastolic pressure-volume relationship regression), load-independent measures of contractility (end-systolic elastance [Ees], the slope of the ESPVR relationship and preload recruitable stroke work [PRSW], and the slope of the linear relationship between stroke work and end-diastolic volume), stroke work (area bounded by the pressure-volume loop), potential energy (LVESP [(ESV − V_0_)/2] − LVEDP [(EDV − V_0_)/4]), pressure-volume area (PVA, the sum of stroke work and potential energy), and ventricular mechanical efficiency (stroke work/PVA) were calculated for each condition. Implant integrity over time was assessed in vitro (Supplemental Methods) and in vivo by contrast-enhanced multiphase computed tomography (SOMATOM Force, Siemens).

### Histology

After euthanasia, histologic samples were collected at points of interest, fixed in 10% formalin, and stained with hematoxylin-eosin and Masson’s trichrome.

### Data analysis

CMR was analyzed using SuiteHeart v5.0.2 (NeoSoft) and computed tomography with 3mensio v10.2 (Pie Medical). Data were analyzed using R v4.1.1 (R Core Team). Continuous variables are presented as median with 25th to 75th percentiles (quartile 1-quartile 3) and categoric variables as counts (percentages). Pre- and postimplant data were compared using Wilcoxon signed rank tests. *P* values <0.05 were deemed statistically significant, although there were no corrections for multiple comparisons in time series.

## Results

### MIRTH technical development, success, and complications

The technique was developed in 18 nonsurvival procedures (Supplemental Results) and then tested in 12 consecutive healthy survival swine procedures. A representative MIRTH procedure sequence is shown in Figure 1. The technique was successful (ie, myocardial entry, controlled intramyocardial navigation around the chamber, intramyocardial ensnarement, implant delivery, and crimp fastener application) in 11 of 12 cases. The sole unsuccessful procedure was from failure of a reused crimp fastener device after an otherwise successful MIRTH implantation. The procedure duration was 110 minutes (104-116 minutes) in the final tercile after “learning.”

Unipolar EDEN electrogram morphology varied depending on the intramyocardial depth of the guidewire tip as depicted in Figure 4 and helped the operator to redirect the guidewire position and orientation during MIRTH navigation. The ST-segment was dramatically elevated for midmyocardial locations, mildly elevated in subendocardial or subepicardial locations, and isoelectric for locations within the heart chambers or pericardium. The QRS complex was indistinguishable from the ST-segment in midmyocardial locations.

Two early implants broke as revealed by computed tomography between days 60 and 90. The failure mechanism was abrasion of standard polyethylene suture, prompting a change to ultra-high molecular weight polyethylene suture. Nine of 12 survived to 90 days; the other 2 died of unrelated bacterial sepsis confirmed at necropsy.

At 90 days, despite aggressive overshortening in growing animals, no MIRTH implant exhibited myocardial erosion. Minor (“protective”) fibrosis surrounded the implants, and the floor of the coronary sinus was fully endothelialized.

### MIRTH-induced changes in left ventricular geometry, structure, and function

In healthy animals, MIRTH implants were tailored to shorten the left ventricular perimeter by an arbitrarily selected one-third based on preprocedure CMR. An immediate reduction in diastolic and systolic chamber dimensions was evident at the level of the implant (Table 1, Figure 3, Supplemental Video 3, Supplemental Video 4). Similar changes were observed in systole and did not vary based on implant location. Chamber reduction was maintained to 90 days in all animals with an intact device.

**Table 1:** Ventricular Cavity Dimensions at the Level of the MIRTH Implant in Healthy Animals

Dimension	Diastolic Diameter (mm)				Systolic Diameter (mm)	
Healthy	Before (n = 11)	After (n = 11)	Day 90^a^ (n = 7)	Before (n = 11)	After (n = 11)	Day 90^a^ (n = 7)
Septolateral	46.3 (43.7-48.8)	29.5^b^ (28.6-31.8)	29.9^c^ (28.0-30.8)	35.5 (31.5-37.6)	27.8^b^ (27.2-28.3)	31.6^c^ (26.9-32.5)
Anteroposterior	46.9 (45.4-48.8)	31.4^d^ (29.6-33.7)	29.7^c^ (26.4-30.9)	32.9 (31.9-34.7)	26.6^b^ (24.6-27.4)	25.9^c^ (23.8-28.9)

Values are median (quartile 1-quartile 3).

MIRTH = Myocardial Intramural Remodeling by Transvenous TeTHer.

a Excluding separated implants.

b *P <* 0.01,

c *P <* 0.05, and

d *P <* 0.001 compared with baseline value using the Wilcoxon signed rank test.

Indexed left ventricular end-diastolic and -systolic volumes were immediately reduced by 13 mL (12%, *P =* 0.005) and 9 mL (16%, *P =* 0.001), respectively, in healthy animals, leading to a 6% increase in left ventricular ejection fraction *(P =* 0.045) (Table 2, Figure 3). MIRTH caused an immediate 25% increase (Δ3 mm Hg, *P =* 0.041) in left ventricular end-diastolic pressure (LVEDP) that normalized during follow-up.

**Table 2:** Structure and Function Measurements Before and After MIRTH in Healthy Animals

		Before (n = 11)	After (n = 11)	Day 90^a^ (n = 7)
LVEDVI, mL/m^2^	Combined	108.2 (104.2-114.8)	**95.5 (93.7-104.5)**^b^	111.6 (104.5-124.8)
	Mid	106.1 (99.9-111.1)	93.9 (90.7-97.7)	111.6 (106.8-114.8)
	Basal	119.6 (107.3-129.7)	103.2 (97.3-110.9)	135.3 (118.8-151.8)
LVESVI, mL/m^2^	Combined	57.5 (53.4-62.3)	**48.1 (44.8-53.5)**^c^	54.4 (44.6-65.3)
	Mid	56.0 (53.4-58.3)	**45.1 (43.2-49.2)**^d^	54.4 (47.3-63.8)
	Basal	65.4 (57.9-70.3)	51.4 (48.3-56.0)	70.3 (56.1-84.5)
LVEF, %	Combined	47 (45-48.5)	**50 (49.5-53.0)**^d^	52 (46-59)
	Mid	47 (45-47.5)	51 (48-55)	52 (50-58)
	Basal	47 (44.3-50.3)	50 (50-50)	50 (46-55)
GLS, %	Combined	−11.4 (−12.2 to −10.1)	−11.5 (−12.7 to −9.5)	−**8.9 (**−**10.2 to** −**7.8)**^d^
	Mid	−10.6 (−11.8 to −9.3)	−9.7 (−11.7 to −8.3)	−9.0 (−9.7 to −8.2)
	Basal	−11.7 (−12.3 to −11.5)	−13.5 (−14.8 to −12.2)	−10.9 (−11.8 to −9.9)
GRS, %	Combined	50.3 (42.2-58.6)	52.6 (50.6-60.7)	50.5 (44.1-56.3)
	Mid	46.0 (37.9-58.6)	51 (47.2-55.2)	44.1 (39.6-48.0)
	Basal	52.9 (49.5-57.1)	60.4 (54.9-66.2)	57.3 (56.7-57.8)
LVEDP, mm Hg	Combined	12 (10-14)	**15 (11-16)**^d^	10 (8-12)
	Mid	12 (10-14)	16 (12-18)	11 (10-12)
	Basal	12 (10-12)	14 (11-15)	9 (9-10)

Values are median (quartile 1-quartile 3).

GLS = global longitudinal strain; GRS = global longitudinal strain; LVEDP = left ventricular end-diastolic pressure; LVEDVI = indexed left ventricular end-diastolic volume; LVEF = left ventricular ejection fraction; LVESVI = indexed left ventricular end systolic volume; MIRTH = Myocardial Intramural Remodeling by Transvenous TeTHer.

a Excluding separated implants.

b *P <* 0.01,

c *P <* 0.001, and

d *P <* 0.05 following comparison to baseline value using the Wilcoxon signed rank test. Values highlighted in **bold** are statistically significant.

**Figure 5:**
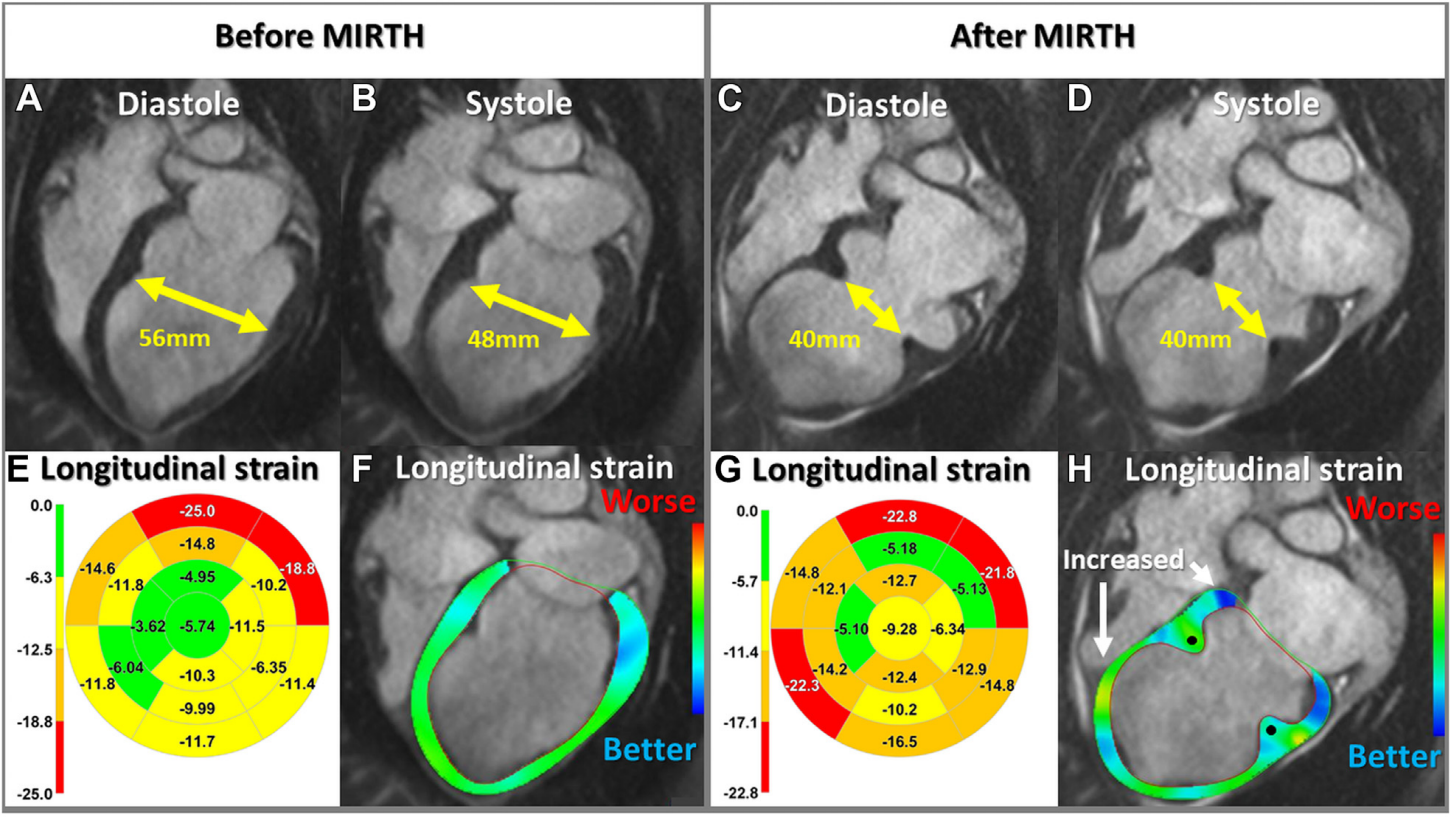
Left Ventricular Morphology and Longitudinal Strain After MIRTH in a Cardiomyopathic Ventricle Four-chamber cardiac magnetic resonance (CMR) **(A and B)** before and **(C and D)** after MIRTH (Myocardial Intramural Remodeling by Transvenous Tether). Left ventricular dimensions are reduced at the level of the implant **(double-headed arrows)** in **(A vs C)** diastole and **(B vs D)** systole. In the same animal, regional longitudinal strain is depicted on **(E and G)** 16-segment bull’s-eye diagrams and **(F and H)** color-overlay CMR strain images at end-systole before and after MIRTH. The midventricular implant improved remote longitudinal strain at the base and ventricular apex and reduced local strain surrounding the implant **(solid circle)**.

By design, MIRTH limited local myocardial strain; it induced an immediate reciprocal favorable increase in remote myocardial strain. This was observed consistently among naive (Figure 2) and cardiomyopathic animals (Figure 5) and was maintained at 90 days. Combined, the net global strain was unchanged.

### Myocardial energetics: dose-response relationship

**Figure 6:**
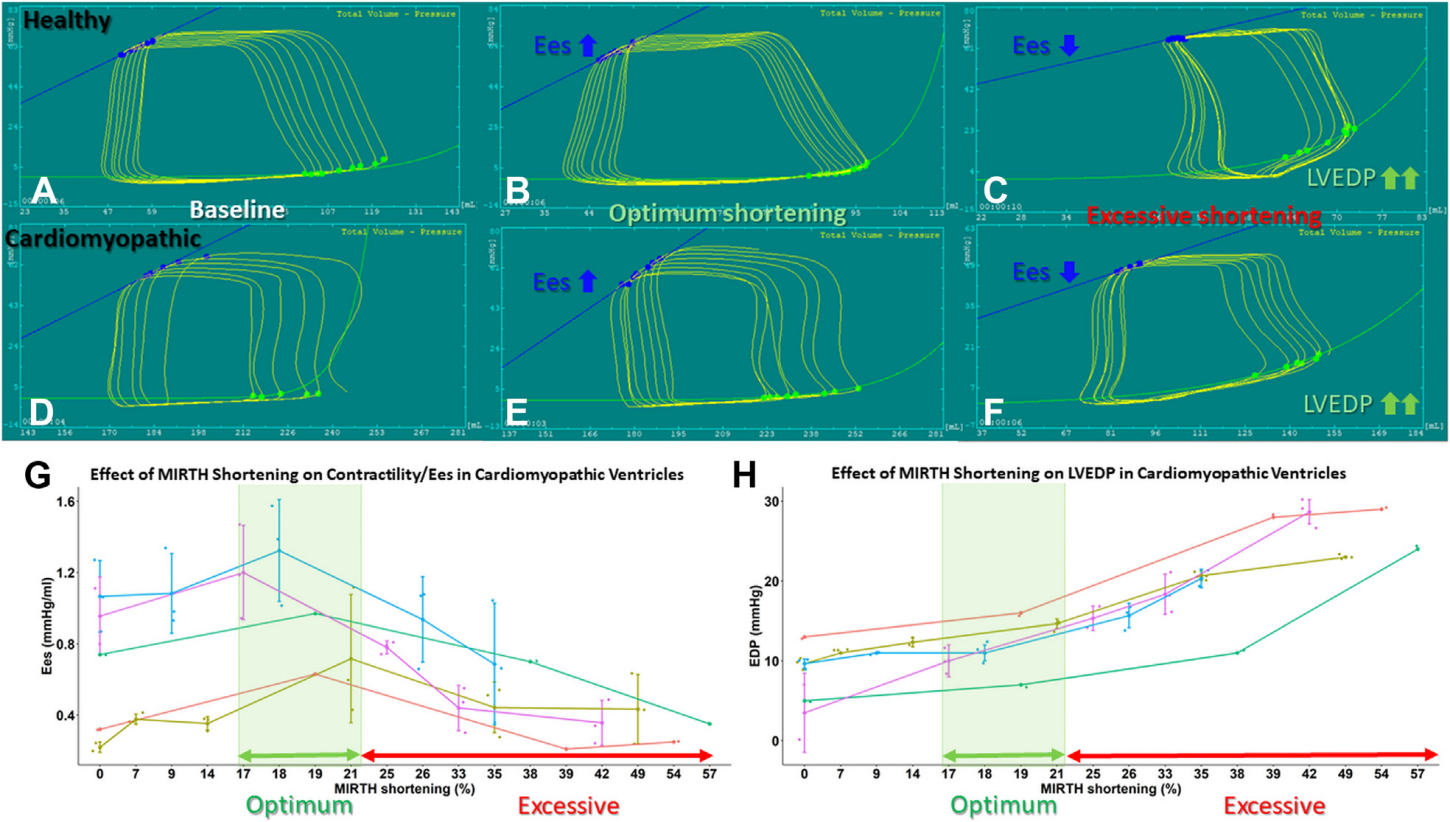
Pressure-Volume Loops Before and Immediately After MIRTH **(A to C)** Healthy and **(D to F)** cardiomyopathic ventricles at **(A and D)** baseline, **(B and E)** with optimum MIRTH (Myocardial Intramural Remodeling by Transvenous Tether) shortening, and **(C and F)** with excessive shortening **(C and F)**. The impact of MIRTH shortening on **(G)** end-systolic elastance (Ees) and **(H)** left ventricular end-diastolic pressure (LVEDP) in cardiomyopathic ventricles. In both healthy and diseased ventricles, progressive MIRTH shortening increased Ees up to a threshold, beyond which additional tension degraded Ees. An inflection in LVEDP is present above 21% MIRTH shortening.

In cardiomyopathic ventricles, there was a biphasic response in myocardial performance to progressive MIRTH circumferential shortening (Figures 6D to 6G). With initial shortening, load-independent measures of contractility (Ees and PRSW) and ventricular mechanical efficiency increased. Other parameters also changed with progressive MIRTH shortening. PVA changed inversely with Ees; PVA changes are proportional to myocardial oxygen demand.^17^^,^^18^ V_o_ changed concordantly with Ees. After a peak, additional shortening degraded performance (Table 3). Importantly, optimum shortening coincided with an inflection in the shortening LVEDP curve (Figure 6H). By contrast, in healthy ventricles, only Ees increased with MIRTH shortening, to a threshold above which additional shortening reduced Ees (Figures 6A to 6C). As expected, other measures of myocardial performance did not improve with MIRTH shortening in healthy ventricles (Table 3). In cardiomyopathic ventricles, the optimum range of MIRTH circumferential shortening that improved indexes of myocardial performance was 17% to 21%.

**Table 3:** Myocardial Energetics (Pressure-Volume Loop Measurements)

Cohort	Shortening (%)	EDV (mL	ESV (mL)	Ees (mm Hg/mL)	EDPVR Slope (mm Hg/mL)	SV (mL)	V_0_ (mL)	Stroke Work (mm Hg/mL)	Potential Energy (mm Hg/mL)	PVA (mm Hg/mL)	Ventricular Mechanical Efficiency (%)	PRSW (mm Hg)
ICM (n = 5)	0	277 (215-325)	198 (140-258)	0.80 (0.32-1.06)	0.006 (0.002-0.015)	73 (67-77)	73 (13-97)	4,600 (3,536-4,674)	3,255 (1,574-7,380)	7,984 (4,813-11,989)	59 (39-67)	30.2 (6.39-47.9)
	9 (7-14)	315 (197-319)	251 (125-252)	0.38 (0.36-0.93)	0.009 (0.009-0.010)	69 (67-69)	70 (67-77)	4,196 (3,054-4,413)	5,915 (1,412-6,284)	10,094 (4,466-10,697)	42 (41-68)	36.6 (35.0-49.0)
	**18**^a^**(17-20)**	**277 (226-314)**	**197 (170-245)**	**1.01 (0.86-1.24)**	**0.010 (0.006-0.022)**	**69 (64-76)**	**115 (85-130)**	**4,013 (2,836-4,446)**	**1,892 (1,499-2,828)**	**5,954 (4,606-7,185)**	**68 (61-71)**	**56.5 (48.0-64.6)**
	33 (26-35)	250 (179-270)	186 (126-192)	0.66 (0.47-0.79)	0.027 (0.014-0.039)	55 (50-65)	54 (29-87)	3,128 (1,924-3,543)	3,199 (1,992-3,807)	6,472 (3,746-7,160)	51 (41-55)	30.1 (4.2-47.0)
	49 (42-51)	243 (226-293)	198 (171-200)	0.35 (0.25-0.45)	0.044 (0.029-0.060)	52 (40-66)	−30 (−50 to 50)	2,630 (2,247-4,033)	3,660 (2,180-6,070)	6,957 (5,727-9,246)	37 (26-55)	13.8 (−11.0 to 28.8)
Healthy (n = 3)	0	109 (106-113)	64 (51-71)	0.93 (0.83-1.28)	0.003 (0.002-0.004)	46 (45-54)	−1 (−25 to 19)	2,745 (2,358-3,418)	1,972 (1,766-2,637)	4,511 (4,330-6,087)	56 (54-61)	45.6 (43.3-51.2)
	10.5 (10-12)	104 (102-109)	60 (53-71)	1.09 (0.97-1.26)	0.006 (0.005-0.006)	42 (39-48)	4 (−14 to 15)	2,553 (2,117-3,047)	1,801 (1,521-2,514)	4,268 (3,719-5,341)	57 (55-58)	36.7 (33.6-39.6)
	21 (20-24)	96 (94-99)	58 (49-68)	0.97 (0.71-1.18)	0.008 (0.006-0.008)	37 (31-43)	−10 (−26 to −1)	1,888 (1,776-2,325)	2,265 (1,701-2,637)	4,336 (4,026-5,069)	51 (40-58)	32.0 (24.3-37.7)
	33.5 (31.5-35)	93 (90-94)	63 (56-70)	0.37 (0.27-0.93)	0.010 (0.007- 0.018)	26 (23-31)	−83 (−133 to −2)	1,342 (1,214-1,862)	4,761 (2,541-6,993)	5,967 (5,073-8,254)	22 (16-52)	18.2 (13.3-25.5)
	47 (42-47)	81 (79-87)	51 (50-58)	0.66 (0.33-0.92)	0.008 (0.006- 0.015)	30 (20-37)	−42 (−75 to −25)	1,662 (636-2,495)	2,847 (2,572-3,709)	4,509 (4,389-4,521)	37 (13-49)	24.9 (21.0-38.6)

Values are median (quartile 1-quartile 3).

EDV = end-diastolic volume; EDPVR = end-diastolic pressure volume relationship; Ees = end-systolic elastance; ESV = end-systolic volume; ICM = ischemic cardiomyopathy; MIRTH = Myocardial Intramural Remodeling by Transvenous Tether; PRSW = preload-recruitable stroke work; PVA = pressure-volume area; SV = stroke volume.

a Optimum MIRTH shortening. Values in **bold** correspond to optimum MIRTH shortening.

### MIRTH navigation through naive and fibrotic myocardium

We tested whether MIRTH-controlled guidewire traversal was feasible and safe through healed myocardial infarction because patients with cardiomyopathy may have fibrosis in regions targeted for MIRTH.

**Figure 7:**
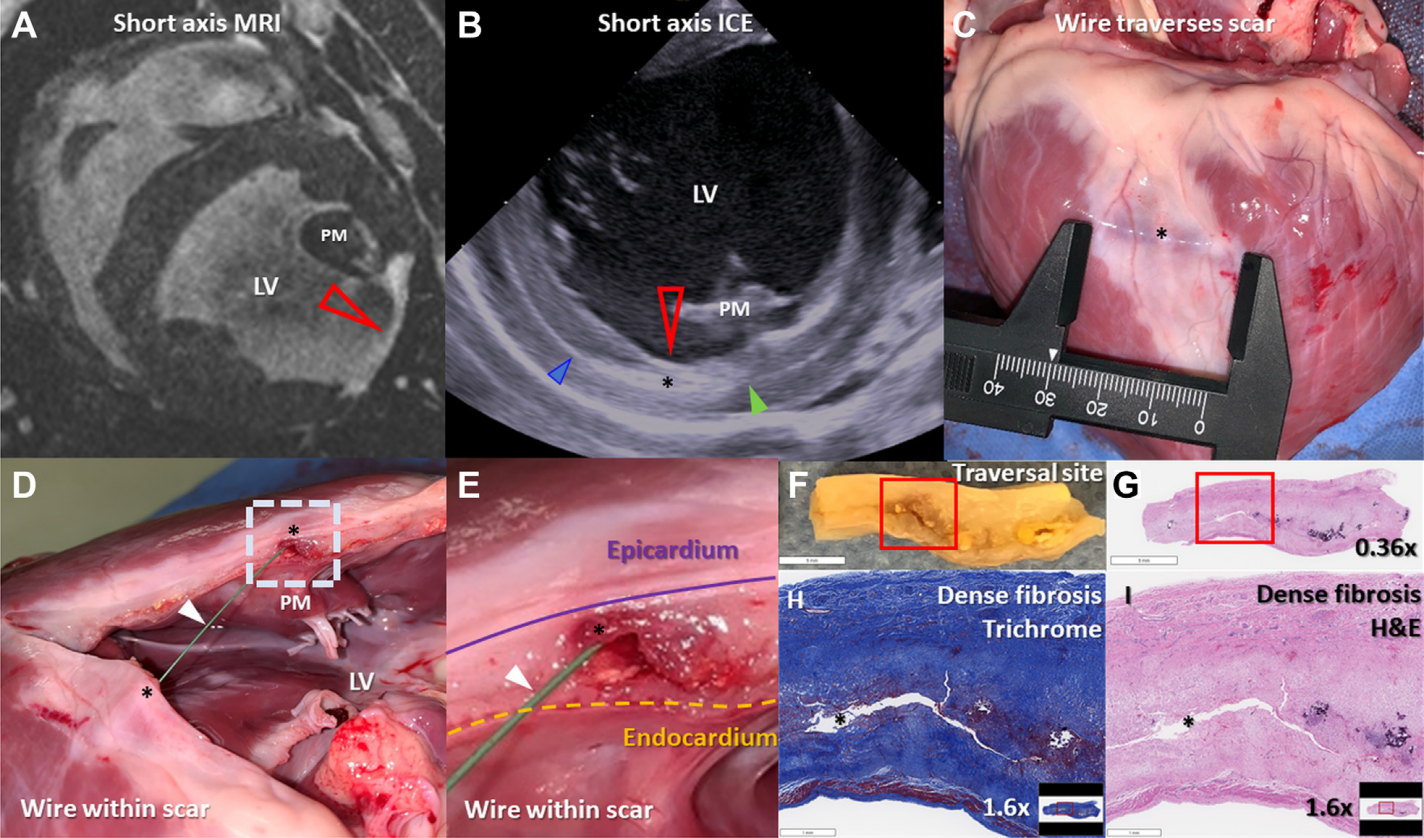
Traversal of Chronic Myocardial Fibrosis Using Intramyocardial Guidewire Navigation **(A)** Short-axis T1-weighted late gadolinium-enhanced cardiac magnetic resonance showing a 3-mm thick region of hyperenhancement in the lateral wall consistent with myocardial fibrosis **(red arrowhead)**. **(B)** Intraprocedure short-axis intracardiac echocardiogram. The MIRTH (Myocardial Intramural Remodeling by Transvenous Tether) guidewire traverses **(black asterisk)** the lateral left ventricular wall entering **(blue arrowhead)** and then exiting **(green arrowhead)** an area of myocardial fibrosis **(red arrow)**. **(C to E)** The explanted heart shows guidewire **(arrowhead)** traversal through dense scar **(black asterisk)**. **(F and G)** Tissue from the area of guidewire traversal in **E** and the corresponding hematoxylin-eosin (H&E)-stained section. Magnified area within the **red box** stained with **(H)** Masson’s trichrome and **(I)** hematoxylin-eosin showing dense replacement fibrosis and no healthy myocardium at the site of guidewire traversal **(black asterisk)**. ICE = intracardiac echocardiography; LV = left ventricular; MRI = magnetic resonance imaging; PM = anterolateral papillary muscle.

MIRTH was accomplished in 13 animals with ischemic cardiomyopathy and involved traversal of 5 to 60 mm of 2- to 3-mm thick myocardial fibrosis 27 days (24-117 days) after infarction (Figure 7). The most mature infarct was 161 days old. EDEN electrograms were diminished or absent within fibrosis; nevertheless, navigation was successful within fibrotic segments relying only on tactile feedback, fluoroscopy, and intracardiac echocardiography.

### MIRTH at base versus midmyocardial level

We tested whether circumferential MIRTH induced different remodeling responses when applied at the midventricular level compared with the basal level (Figure 2). Basal and mid MIRTH implants reduced left ventricular chamber volumes comparably on CMR (Table 2). The net global function by all measures of strain (longitudinal, radial, and circumferential) was also similar *(P =* NS). By contrast, regional strain analysis revealed remote increases in the base and apex for midventricular implants and in the mid and apical ventricle for basal implants (Figure 2).

Because midmyocardial anatomical remodeling can have a disproportionate functional impact on the mitral valve via altered papillary geometry and chordal tension, we measured the impact on the interpapillary distance from both basal and midventricular MIRTH trajectories. Midmyocardial MIRTH reduced the interpapillary distance, whereas basal MIRTH did not (Table 4, Figure 3C).

**Table 4:** Interpapillary Distance on Magnetic Resonance Imaging Before and After MIRTH in Healthy Animals

	Diastolic Diameter (cm)			Systolic Diameter (cm)		
	Before	After	Day 90^a^	Before	After	Day 90^a^
Combined (n = 11)	27.6 (27-29.1)	**20. 5**^b^ (18.2-25.7)	**16.9**^b^ (16.1-23.8)	18.2 (17-19.7)	**15.1**^c^ (12.3-17.4)	12.8 (8.8-20)
Basal only (n = 4)	29 (27.2-30.9)	28.6 (26.2-31.6)	28.2 (27.8-28.6)	19.3 (17.8-20.6)	18.2 (17.5-18.9)	26.2 (25.8-26.5)
Midventricular only (n = 7)	27.6 (27.0-28.5)	**18.9**^b^ (16.3-19.8)	16. 4 (15.7-16.9)	17.6 (16.7-19.2)	**12.7**^b^ (11.0-15.1)	9.7 (7.8-12.8)

Values are median (quartile 1-quartile 3).

Abbreviation as in Tables 1 and 2.

a Excluding separated implants.

b *P <* 0.05 and

c *P <* 0.01 compared with baseline value using the Wilcoxon signed rank test. Values in **bold** are statistically significant.

## Discussion

We describe MIRTH, a fully percutaneous, transvenous, regional, circumferential, left ventricular remodeling procedure. The key novelties are controlled intramyocardial navigation and the introduction of a myocardial remodeling device within the walls of the beating left ventricle using clinical off-the-shelf catheter tools. MIRTH offers the following attractive features: 1) MIRTH distributes the centripetal load uniformly within the myocardial wall, unlike anchor-based remodeling devices that focus load discretely at anchor locations; 2) MIRTH shortening achieves a predictable reduction in left ventricular chamber dimensions that is sustained for the months-long duration of testing without endomyocardial erosion; 3) MIRTH shortening favorably increases load-independent measures of myocardial contractility (Ees and PRSW) in a dose-dependent manner up to a maximum, beyond which additional shortening unfavorably reduces contractility and causes end-diastolic pressure to rise; 4) because it is a regional rather than a global myocardial remodeling device, MIRTH does not appear to have a deleterious effect on ventricular compliance; 5) MIRTH implants can be positioned at will (eg, at the basal or midventricular level); and 6) at a midventricular level, MIRTH approximates papillary muscles, which may reduce leaflet traction contributing to functional mitral regurgitation.

### Intramyocardial guidewire navigation and EDEN unipolar electrogram tracking

The MIRTH procedure introduces several novel techniques including: 1) controlled intramyocardial guidewire navigation; 2) EDEN unipolar electrogram guidance; 3) a transmural anchor guidewire into the left ventricular cavity inspired by Khelimskii et al^19^ to stabilize catheter equipment during the procedure; 4) guidewire ensnarement within the myocardial wall; and 5) implantation and tensioning of an intramyocardial shortening device.

In MIRTH, we steer a commercially available angioplasty guidewire freely within the walls of the left ventricle. After the addition of a 30° to 40° CTO tip bend, and once delivered to the ventricular myocardium, a combination of forward and rotational movements grants the ability to “navigate” with multiple degrees of freedom. We show that wires can be steered at will to accomplish any desired intramyocardial position (eg, from base to apex, apex to base, endocardium to epicardium, epicardium to endocardium, and obliquely in either direction and at any depth) irrespective of myofiber orientation.^20^ We modified this guidewire technique from transcatheter mitral cerclage^11^^,^^21^ wherein we traverse the interventricular septum with a guidewire from a septal perforator vein into the right ventricular infundibular cavity. In our U.S. feasibility study,^22^ we observed that a CTO angulated tip afforded great versatility in septal guidewire navigation. Such guidewire manipulation does not recognizably injure the myocardium or induce epicardial bleeding.

Identifying the guidewire tip position has proven difficult using conventional imaging modalities, which suffer from poor soft tissue definition, off-axis imaging planes, and poor intramyocardial device tracking. We developed the EDEN technique as a simple way to continuously ensure the operator of an appropriately deep intramyocardial position. Using the uninsulated guidewire tip as a unipolar electrode, we characterized local unipolar electrogram morphology that distinguishes the desirable position (deep within the myocardial wall) from undesirable positions too close to the endocardial or epicardial surfaces. When subepicardial or subendocardial electrograms are encountered, the operator simply withdraws and redirects the guidewire until a deep myocardial signal is identified. This is analogous to a coronary guidewire operator withdrawing and redirecting a guidewire from an undesirable side branch.

Finally, we were able to accomplish guidewire navigation and traversal even through fibrotic, chronically infarcted, and thinned (3 mm) myocardium using tactile feedback and imaging guidance even when the EDEN electrogram amplitude was attenuated or absent.

An intracavitary anchor guidewire (from the coronary sinus and across the posterobasal myocardium into the left ventricle) permitted efficient equipment exchanges and delivery of a stent-retriever snare through a single access point. This new technique may have other applications beyond MIRTH when percutaneous access to the left ventricular myocardium is otherwise unavailable.

### Myocardial remodeling

Ventricular wall stress is a major determinant of myocardial oxygen consumption.^23^ Global and segmental left ventricular remodeling therapies aim to reduce ventricular diameter and, via Laplace principles, wall stress. Like both Coapsys^24^ and Myosplint^25^ devices, we demonstrate that MIRTH constrains wall motion locally where it reduces cardiac dimensions and conversely increases regional strain (and contractility) elsewhere in the heart. MIRTH placed in the base of the heart increases midventricular and apical strain. Similarly, MIRTH placed in the midventricular position increases basal and apical strain.

We show that in cardiomyopathic ventricles MIRTH exhibits a biphasic dose response in ventricular performance and myocardial energetics. Progressive MIRTH circumferential shortening improves Ees and PRSW (as load-independent measures of contractility) and ventricular mechanical efficiency up to a maximum, beyond which additional MIRTH shortening causes these measures to decline. In addition, PVA, which is directly proportional to myocardial oxygen demand,^17^^,^^18^ decreases to the same threshold. These concordant findings suggest MIRTH offers potential benefit within a therapeutic window by reducing remote wall tension with its attendant reduction of myocardial oxygen demand. Improvements in several measures of contractility were concordant, such as Ees, PRSW, and ventricular mechanical efficiency. Ees and V_0_ have a complex relationship that has been reviewed by Burkhoff et al^26^ increased contractility may manifest in several ways, including by concordant changes in Ees and V_0_, similar to that observed in our cardiomyopathic animals. Importantly, we observed an inflection in LVEDP increase at optimum MIRTH shortening, which can serve as a useful and easily measured parameter to titrate shortening during implantation in patients.

We observed no late myocardial erosion of MIRTH implants in a model that may increase erosion propensity; these rapidly growing juvenile swine increased myocardial mass by 175%.

MIRTH addresses limitations of other ventricular remodeling approaches. It broadly redistributes the centripetal load to avoid avulsion and device fractures observed in endocardial anchor-based devices such as AccuCinch (Ancora Heart).^27^ It averts the morbidity of reconstructive surgery^2^ or transthoracic implants.^3^, ^4^, ^5^, ^6^, ^7^, ^8^ As a transvenous percutaneous procedure, there is no left ventricular blood contact, in contrast to endocavitary excluder devices.^9^ MIRTH can be implanted without regard to anatomical structures (eg, mitral structures, epicardial coronary arteries, and His bundle) or tissue characteristics (eg, local fibrosis or scar). MIRTH does not constrain the right ventricle.

### Study limitations

We tested only commercial off-the-shelf clinical catheter tools rather than purpose-built devices that may be easier to use and may prove more durable. Our abbreviated fatigue testing does not meet contemporary standards for cardiovascular implants.^28^^,^^29^ Our statistical analysis did not correct for multiple comparisons, so *P* values should be interpreted with caution. The sample size for animals undergoing pressure-volume loop analysis was small.

We await survival tests of appropriate MIRTH shortening in animal models of cardiomyopathy. It is possible that ischemic cardiomyopathy, pacing-induced cardiomyopathy, or toxic cardiomyopathy models may not predict MIRTH performance in patients.

## Next Steps and Other Applications

MIRTH may have value in the treatment of dilated cardiomyopathy. MIRTH may also attenuate unfavorable remodeling after acute myocardial infarction. Purpose-built devices would be desirable for clinical investigation.

Intramyocardial navigation is also a key step in the Septal Scoring Along Midline Endocardium technique of transcatheter ventriculomyotomy for left ventricular outflow obstruction^30^ recently applied to patients.^31^ Intramyocardial positioning of an insulated guidewire may also have value in focal ablation of poorly accessible targets currently treated with extensive radiofrequency ablation or chemoablation (eg, at the basal interventricular septum [“summit”] or the base of the papillary muscles^32^).

## Conclusions

We describe MIRTH, a novel transcatheter myocardial remodeling device and strategy, in animals. Intramyocardial guidewire navigation and EDEN intracardiac electrogram guidance are completely new concepts that may inspire and enable new cardiovascular interventions. Clinical investigation is warranted.

Perspectives **COMPETENCY IN MEDICAL KNOWLEDGE:** Patients with dilated cardiomyopathy who fail to respond to optimal medical therapy and fail to respond to or are ineligible for cardiac resynchronization therapy have few options short of ventricular assist devices and heart transplantation. Mechanical heart failure therapies may fill this unmet clinical need and delay, reverse, or prevent the progression of left ventricular dilation.

**TRANSLATIONAL OUTLOOK:** Controlled intramyocardial guidewire navigation is a new procedural technique that permits access to any location within the ventricular myocardium. Notoriously inaccessible locations in the basal interventricular septum (“summit”), base of the papillary muscles, or deep within the ventricular walls are now accessible percutaneously using commercially available devices for interventional electrophysiological and structural heart procedures.

## Funding Support and Author Disclosures

This work was supported by the Division of Intramural Research, National Heart Lung and Blood Institute, National Institutes of Health USA (grant Z01-HL006040 to Dr Lederman). Drs Bruce and Lederman are coinventors on patents, assigned to National Institutes of Health, on MIRTH-related devices. Dr Rogers is a consultant and physician proctor for Edwards Lifesciences and Medtronic; is a Medtronic advisory board member; and has an equity interest in Transmural Systems. Drs Babaliaros and Greenbaum receive institutional research support from Abbott Vascular, Ancora Heart, Edwards Lifesciences, Gore Medical, Jena Valve, Medtronic, Polares Medical, Transmural Systems, and 4C Medical; receive consulting fees from Abbott Vascular, Edwards Lifesciences, and Medtronic; and have equity interest in Transmural Systems. Drs Campbell-Washburn, Herzka, and Lederman are investigators on a U.S. Government Cooperative Research and Development Agreement with Siemens. Siemens participated in the modification of the MRI system from 1.5-T to 0.55-T and provided investigational intracardiac echocardiography devices. All other authors have reported that they have no relationships relevant to the contents of this paper to disclose.

## Appendix

Supplemental Data

Supplemental Video 1 Creating a MIRTH Loop

Supplemental Video 2 Delivering a MIRTH Implant

Supplemental Video 3 Left Ventriculogram Before and After MIRTH

Supplemental Video 4 Controlled Intramyocardial Navigation

Supplemental Video 5 Volume-Rendered CT of MIRTH Implant 30 Days After Implantation
