# Supplementary material for: Reshaping the Ventricle From Within: MIRTH (Myocardial Intramural Remodeling by Transvenous Tether) Ventriculoplasty in Swine
Source: JACC Basic Transl Sci. 2022 Nov 9;8(1):37–50. doi: 10.1016/j.jacbts.2022.07.002 (PMC9911325; doi:10.1016/j.jacbts.2022.07.002)
Supplement: Supplemental Data [file mmc1.pdf]

SUPPLEMENTAL METHODS

Study flow

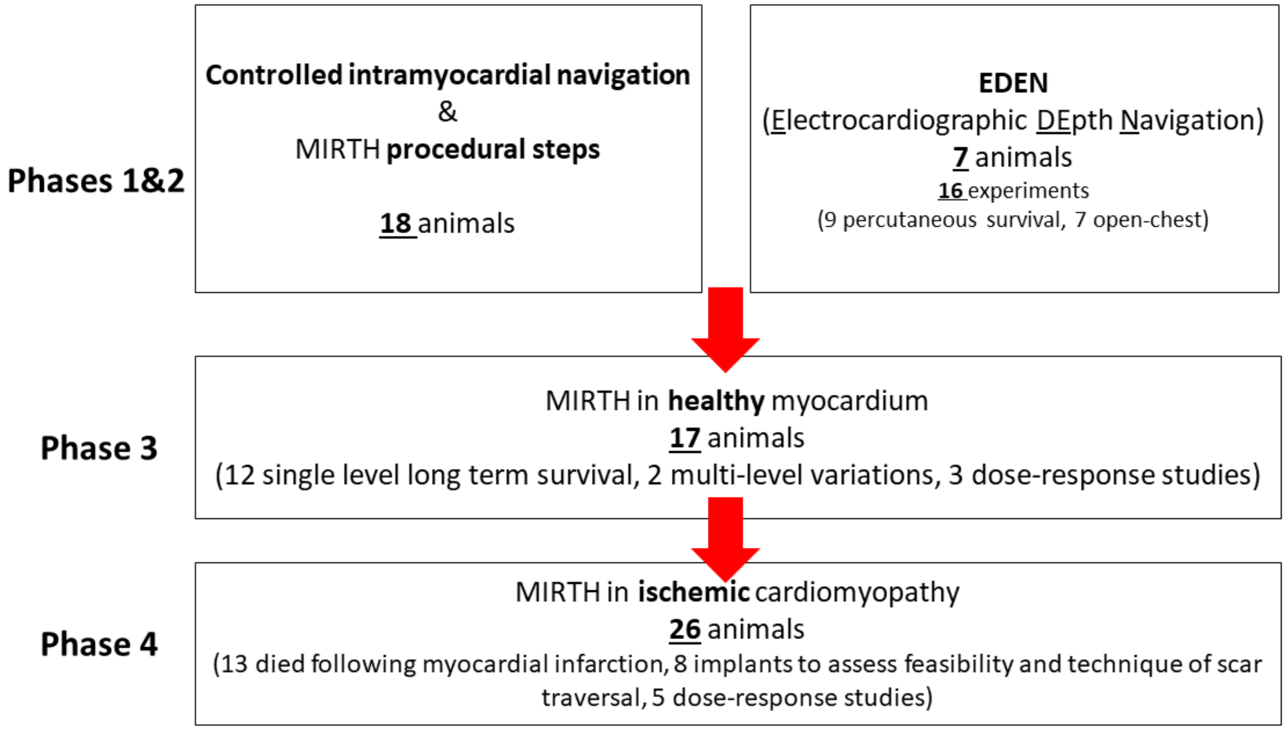

MIRTH technical refinements during early experimentation

Technical refinement included (a) care to assure the trajectory remains within the myocardium during ensnarement, using EDEN electrogram and ICE guidance; (b) assuring complete microcatheter ensheathment of the implant suture to prevent abrasion against the crimp-fastener over time; and (c) change from polyethylene (*Ethibond*, Ethicon) suture to ultra-high molecular weight polyethylene (UHMWPE) suture (*HS Fiber*, Riverpoint Medical) that better resists abrasion.

EDEN (Electrocardiographic radial DEpth Navigation) experiments

Preliminary experiments

In 9 percutaneous experiments we performed electrogram mapping from the left and right ventricles and interventricular septum using the same guidewire and microcatheter combinations used in MIRTH; in 7 open-chest, non-survival experiments we used a custom calibrated multi-electrode plunge needle.

MIRTH implant *in-vitro* fatigue testing

In vitro implant fatigue testing

To evaluate implant resistance to repetitive stress beyond which we could achieve in animal models, MIRTH implants were fixed to a custom jig generating cyclic expansion between 0.5-5 pounds/in^2 (3.5 – 35kPa) at 1000 cycles/minute. Implants were evaluated at intervals for signs of erosion and separation up to 20 million cycles.

SUPPLEMENTAL RESULTS

Fatigue testing

Four model implants (2 ultra-high molecular weight polyethylene, 2 polyethylene) secured with a single surgical crimp-fastener failed due to crimp slippage after 7- 9 million cycles. Interposing a Roeder knot between the MIRTH tether and crimp allowed four implants to complete the 20 million cycle test without failure.
